# Carcinome basocellulaire à localisation bilatérale de traitement curatif simple

**DOI:** 10.11604/pamj.2016.25.168.11011

**Published:** 2016-11-16

**Authors:** Karim Bourra, Adil Arrob

**Affiliations:** 1 Service de Chirurgie Plastique Hôpital Al Farabi Oujda; 2 Service Chirurgie Plastique Hôpital d’Instruction Militaire Mohammed V Rabat

**Keywords:** CBC, cancer cutané, greffe de peau total, lésions perlées, Basal cell carcinoma, squamous cell carcinoma, leishmaniasis

## Image en médecine

Il s'agit d'un cas très rare de carcinome basocellulaire (CBC) des paupières inférieures à doubles localisations (A) apparu de manière progressive d'aggravation lente secondaire à une exposition massive au soleil accumulée probablement durant l'enfance vu que le patient est un paysan habitant en milieu rural avec notion d'exposition solaire habituelle et sans protection solaire par crèmes solaires ou autre et ce pendant 25 ans. Actuellement le patient âgé de 58 ans et père de 4 enfant habitant Taroudant au Maroc originaire d'une région enclavée et montagneuse. Le patient ayant bénéficié d'un bilan pré opératoire complet biologique et radiologique avec radio du crâne face et profil revenus normaux ainsi qu'une biopsie partielle de chaque lésion (A) et qui a confirmé la présence d'un CBC cutané développé au dépends de la paupière inférieure et à localisation bilatérale. Nous avons décidé de faire une exérèse du CBC simple avec une marge de sécurité de 1 mm. La lecture Anapath est revenue positive confirmant le diagnostic de CBC cutané à développement local avec des marges d'exérèses saines et même en profondeur. L'intérêt de cette publication est de faire montrer un aspect clinique caractéristique d'un épithélioma basocellulaire avec les fameux “perles épithéilomateuses” (A, C) caractéristiques et confirmés par les examens anatomopathologiques. Le patient fut traité dans un deuxième temps opératoire par greffe de peau totale (B, D) prélevée sur les deux creux sus claviculaires. Le suivi du patient par la suite a montré une guérison totale sans récidive puisque les lésions ont été enlevées en totalité de manière respectant les unités esthétiques des paupières inférieures (B,D).

**Figure 1:**
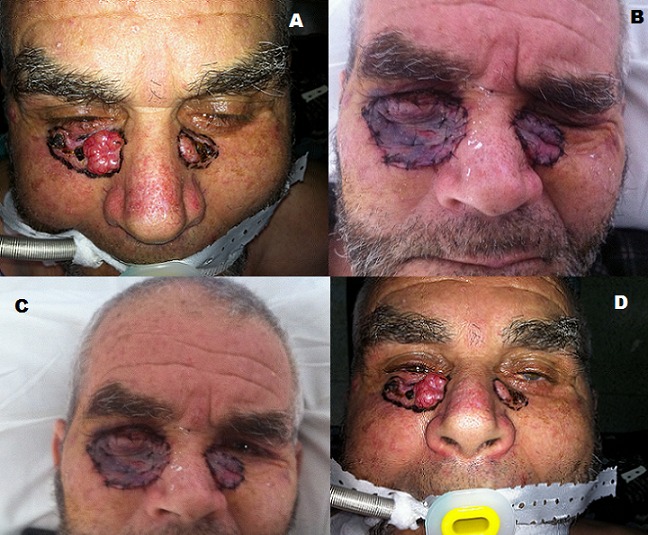
A) CBC des 2 paupières inferieures; B) CBC a localisation bilatérale; C) GPT greffe de peau totale en unité esthétique paup inf; D) Greffe de peau total respect du bord libre paupière inferieures

